# The American Society of ExtraCorporeal Technology Standards and Guidelines for Pediatric and Congenital Perfusion Practice: 2025 Update☆

**DOI:** 10.1051/ject/2026012

**Published:** 2026-09-15

**Authors:** Molly Elisabeth Oldeen, Carrie Whittaker Striker, Ronald Angona, Dafne Andrea Chianella, Chelsea Capone, Ashley B Walczak, Thomas Klein

**Affiliations:** 1 Department of Cardiovascular Perfusion, Norton Children’s Hospital Louisville KY USA; 2 Department of Cardiothoracic Surgery, Division of Cardiovascular Perfusion, Herma Heart Institute Children’s Wisconsin Milwaukee WI USA; 3 Department of Surgery, University of Rochester Rochester NY USA; 4 Department of Cardiovascular Perfusion, Shawn Jenkins Children's Hospital, Medical University of South Carolina Charleston SC USA; 5 Department of Pediatric Perfusion and ECMO Services, Doernbecher Children’s Hospital, Oregon Health and Science University Portland OR USA; 6 Department of Cardiovascular Perfusion, Riverside Methodist Hospital Columbus OH USA; 7 Department of Surgery, Division of Pediatric Cardiothoracic Surgery, University of California Davis Sacramento CA USA

**Keywords:** Congenital heart disease, Cardiopulmonary bypass, Pediatric, Perfusion, Standards, Practice guidelines

## Abstract

*Background*: In 2019, the American Society of ExtraCorporeal Technology (AmSECT) approved the inaugural *Standards and Guidelines for Pediatric and Congenital Perfusion Practice*. These standards and guidelines were created with the intent of periodic revision to ensure continued alignment with evolving best practices. In 2023, an AmSECT subcommittee initiated this review in consideration of current literature and contemporary clinical practices. *Methods*: The subcommittee, consisting of pediatric and congenital perfusionists, conducted a systematic literature review assessing each standard and guideline to determine if current evidence supports elevation of guidelines to standards, incorporation of new guidelines or standards, or whether existing standards and guidelines should remain unchanged. AmSECT’s adult *Standards and Guidelines for Perfusion Practice (2023)* updates were also considered. Proposed revisions were reviewed by the 2024 AmSECT International conference attendees, AmSECT Pediatric and Congenital Perfusion Committee, AmSECT Fellows of Pediatric Perfusion (FPP), and the AmSECT International Consortium for Evidence-Based Perfusion (ICEBP). *Results*: Regarding pediatric and congenital specific changes, five guidelines were elevated to standards and three new guidelines and one standard were introduced. Additionally, five patient safety-related standards and one additional guideline were adopted from the *Standards for Perfusion Practice (2023)* document. *Conclusion*: Over the course of two years, consisting of an extensive literature review and feedback from multiple stakeholders, the 2025 update to the *Standards and Guidelines for Pediatric and Congenital Perfusion Practice* were approved by AmSECT leadership, ratified by an AmSECT membership vote, and subsequently endorsed by CHSS.

## Introduction

Standards and Guidelines provide a framework for best practices, quality improvement, and methodology to reduce patient harm [1]. National organizations such as the Centers for Disease Control (CDC), World Health Organization (WHO), The Joint Commission (TJC), the Institute of Medicine (IOM), and the Occupational Safety and Health Administration (OSHA) are a few organizations that implement, regulate, and promote the use of standards to improve patient safety and outcomes. Specific to cardiac surgery, the Society of Thoracic Surgeons (STS), the Society of Cardiovascular Anesthesiologists (SCA), and the American Association of Thoracic Surgeons (AATS) have clinical practice guidelines that they craft and endorse as a profession [2–4]. While these standards and guidelines are of value, they do not directly address perfusion practices.

The first guidelines for perfusion practice were published in 1993 by the American Society of Extracorporeal Technology (AmSECT) [5]. More recently, the International Consortium for Evidence-Based Perfusion (ICEBP), in collaboration with AmSECT, updated these guidelines in 2013, 2017, and 2023 and are known as *Standards and Guidelines for Perfusion Practice* and endorsed by the STS, SCA and AATS [6]. This document, although foundational, does not adequately address the physiological and technical considerations inherent in pediatric and congenital perfusion practice. Recognizing this critical gap, AmSECT commissioned the development of pediatric-specific standards and guidelines for perfusion practice. With the support of AmSECT’s Pediatric and Congenital Perfusion Committee (PCPC), a subcommittee was tasked with the development of a comprehensive, evidence-based document uniquely tailored to the complex and nuanced needs of pediatric and congenital cardiac patients [7, 8]. These efforts culminated in first-ever *Standards and Guidelines for Pediatric and Congenital Perfusion* and were ratified and published in 2019. Endorsements from the Congenital Heart Surgeons’ Society (CHSS) and the Congenital Cardiac Anesthesia Society (CCAS) were obtained.

Prior to 2019, there were no official documents to guide the practice of pediatric perfusion worldwide. This publication marked a significant advancement in standardizing care, enhancing patient safety, and reducing practice variability in pediatric perfusion. In 2021, an international survey of perfusion practices for pediatric and congenital heart survey revealed that 98% of centers in North America adopted these standards and guidelines indicating that the document offered significant clinical implications [9]. To ensure continued alignment with current research, evidence-based practices, and clinical innovations periodic revision of the standards and guidelines was necessary. In 2023, an AmSECT PCPC subcommittee initiated this process.

## Materials and methods

The process for updating the *Standards and Guidelines for Pediatric and Congenital Perfusion (2019)* commenced following the release of the newly updated adult *Standards and Guidelines for Perfusion Practice document* in 2023. The AmSECT PCPC appointed subcommittee consisted of pediatric and congenital perfusionists from varying institutions across the United States and were chosen specifically for their expertise and background in the field. The subcommittee’s primary objective was to evaluate the latest evidence, gather input from key stakeholders, and provide guidance to perfusionists conducting pediatric and congenital cardiopulmonary bypass (CPB) through an updated standards and guidelines document.

The review process began with assessing the updates made to the adult *Standards and Guidelines for Perfusion Practice (2023)* and to evaluate their applicability to pediatric and congenital perfusion. After that, each section of the *Standards and Guidelines for Pediatric Practice (2019)* was individually evaluated and reviewed to determine if the current evidence and practice trends suggest changes or additions to the existing document. These sections are staffing, communication and hand-off, medical records, checklists, temperature management, anticoagulation, arterial blood pressure, blood gas management, hematocrit expectations, technology updates, cerebral and somatic oximetry, indexed oxygen delivery and consumption, ventilation strategies, blood flow targets, CPB circuitry, CPB circuit priming techniques, blood product management, ultrafiltration, and quality improvement.

When evaluating the literature, an emphasis was placed on journal articles published between 2019 and 2024 to support existing and new recommendations. Inclusion criteria consisted of peer-reviewed pediatric and cardiac surgery-specific publications only. No animal studies were included. Although the literature review prioritized recent research, key references over ten years old were retained when newer evidence was lacking. Any relevant new literature or literature overlooked previously was presented and discussed by the subcommittee as well. After detailed analysis of over 140 publications and committee consensus, the most up-to-date publications and research were added to support recommendations for the revised pediatric and congenital standards and guidelines.

**Figure 1:**
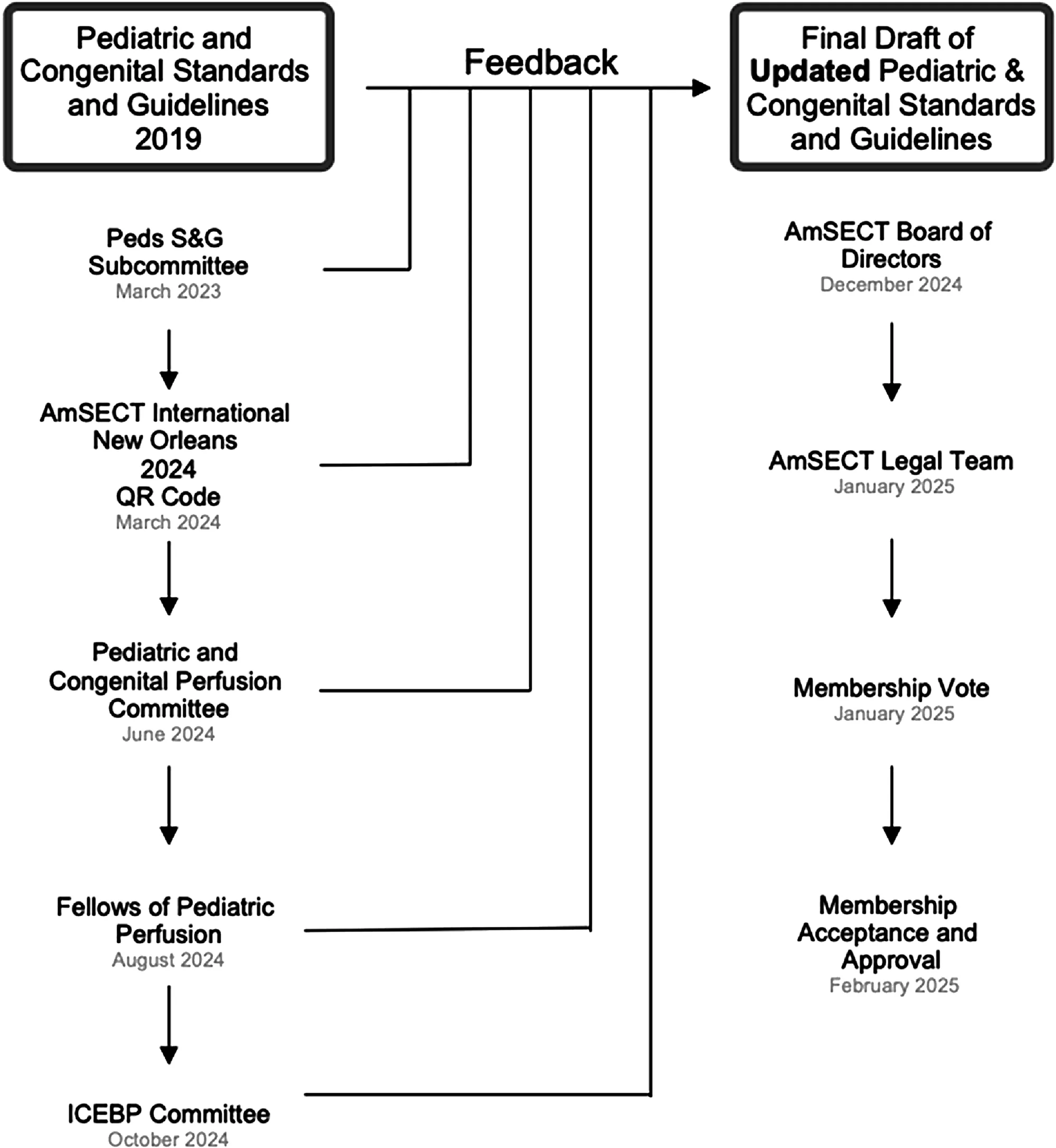
Review and approval process for the revision of the *Standards and Guidelines for Pediatric and Congenital Perfusion Practice.* AmSECT, American Society of ExtraCorporeal Technology; ICEBP, International Consortium for Evidence-Based Perfusion.

A draft of the updated standards and guidelines with highlighted changes was presented to multiple parties for comment. This process in its entirety is depicted in Figure 1. Initially, the draft standards and guidelines were presented in March of 2024 to pediatric perfusionists attending the AmSECT International Meeting in New Orleans, LA. Feedback was captured by providing a code to attendees which directed them to a survey. The same draft revision of the standards and guidelines was submitted to the AmSECT PCPC committee in June 2024 for review. Additionally, in August 2024, a survey was sent to the Fellows of Pediatric Perfusion (FPP) listserv for feedback and in October 2024 to the ICEBP committee. All feedback was reviewed and considered by subcommittee members and incorporated into the final draft where applicable.

**Table 1:** Summary of current *Standards and Guidelines for Pediatric and Congenital Perfusion Practice (2025)* document and highlighted modifications.

**Standard 1: Development of Institutionally Based Protocols**
✓ Access to emergency protocols during an event
**Standard 2: Qualification, Competency and Support Staff**
✓Addition of more detailed onboarding process
**Standard 3: Communication**
✓+ Communication of deviation from treatment plan
✓ Post-procedure debrief
**Standard 4: Perfusion Record**
**Standard 5: Checklist**
**Standard 6: Safety Devices**
**Standard 7: Monitoring**
+ Continuous monitoring of arterial oxygen saturation
**Standard 8: Anticoagulation**
+ Identification of and management of heparin resistance
**Standard 9: Gas Exchange**
* Utilization of pH stat
+ Point of Care Testing usage
**Standard 10: Blood Flow**
*Higher blood flow rates for pediatrics, DO_2_i requirements
**Standard 11: Blood Pressure**
**Standard 12: Circuitry**
**Standard 13: Priming**
*Addition of dilutional hematocrit and osmolarity/osmolality to priming composition considerations
+ Use of pre-bypass ultrafiltration or washing for blood priming
**Standard 14: Protamine and Cardiotomy Suction**
**Standard 15: Blood Management**
*Contribution of hemoglobin to DO_2_i
✓Ultrafiltration to Reduce hemodilution
*Red blood cell washing (cell saver or blood bank)
**Standard 16: Fluid Management**
**Standard 17: Level of Readiness**
✓Circuit assembly and maintenance regulation according to institutional protocol using aseptic technique
**Standard 18: Staffing**
**Standard 19: Duty Hours**
**Standard 20: Quality Assurance and Improvement**
✓+ Collection of data via clinical registry or database
**Standard 21: Maintenance**
**Standard 22: Crisis Management**
✓ 1 Standard and 5 Guidelines

*: Denotes new pediatric specific standards and guidelines; + : Indicates guideline moved to standard; ✓ : Updated from adult *Standards and Guidelines for Perfusion Practice (2023)*.

Once the final draft was composed, it was presented to the AmSECT Board of Directors followed by the AmSECT legal team. After a membership vote in January 2025, the AmSECT membership approved the revised *Standards and Guidelines for Pediatric and Congenital Perfusion Practice (2025)*. This document was endorsed by surgical peers from the Congenital Heart Surgeons’ Society (CHSS) in October 2025.

## Results

As a result of this review, the subcommittee accepted many of the updates to the adult *Standards and Guidelines for Perfusion Practice (2023)* document. These updates encompassed revised definitions, consolidation of content, improved clarity, rewording of multiple standards and guidelines, and the inclusion of new ones. Many of these inclusions are centered around patient safety which transcends patient size or age. The adopted standards and guidelines from the *Standards and Guidelines for Perfusion Practice (2023)* included:

**Standard 1.3**: Perfusion emergency protocols shall be accessible to help guide the perfusionist during an event.
**Standard 3.4**: Deviations from the intended treatment care plan shall be appropriately communicated to the supervising physician and documented to allow for changes in the management plan. (Guideline in Adult)
**Guideline 3.2**: Topics that should be considered during the post-procedure debrief include, but are not limited to, communication, additional training, equipment or disposables issues, post-operative instructions, and safety events.
**Standard 15.1**: The Perfusionist shall utilize the timely and collaborative application of evidence-based medical and surgical concepts (see Guideline 15.1) designed to maintain hemoglobin concentration, optimize hemostasis, and minimize blood loss in an effort to improve patient outcome. (Reworded) (Standard 13 in Adult)
**Standard 17.4**: Assembly and maintenance of circuit shall be regulated according to institutional protocol using aseptic technique.
**Standard 22**: Crisis Management (Standard 20 in Adult).

The addition of Standard 22: Crisis Management arose from the COVID-19 pandemic. Standard 22.1 states, “The perfusionist shall participate in a collaborative effort to implement an actionable crisis management plan for unforeseen circumstances that may prohibit the ability to perform standard duties” [6]. In the setting of the recent pandemic and persistent natural disasters, this standard reminds perfusion teams to prepare for issues related to supply chain, equipment shortages, and clinical and storage availability during these events.

The updated versions for both the *Standards and Guidelines for Perfusion Practice (2023)* and the *Standards and Guidelines for Pediatric and Congenital Perfusion Practice (2025)* include the defined usage of the word continually and continuously. The word “continuously” describes an action that occurs without ceasing, and the word “continually” describes an action that occurs frequently or regularly. Given the exacting nature of caring for pediatric and congenital patients on cardiopulmonary bypass, six standards and guidelines that previously suggested a practice to be done continually, were elevated to continuous or without ceasing. Examples include monitoring of hemoglobin/hematocrit, arterial and venous oxygen saturation, and cerebral and somatic oximetry while on cardiopulmonary bypass. Continuous surveillance of critical parameters highlights the imperative for pediatric and congenital perfusionists to administer care that is both consistent and precise. This obligation is further substantiated by advancements in medical technology which facilitate the continuous monitoring of patient physiological parameters.

Regarding pediatric-specific updates to the *Standards and Guidelines for Pediatric and Congenital Perfusion Practice*, five guidelines were elevated to standards:

**Standard 7.12**: Arterial oxygen saturation shall be monitored continuously during cardiopulmonary bypass procedures.
**Standard 8.2**: A process shall exist for identifying and managing heparin resistance.
**Standard 9.4**: Point-of-Care testing shall be utilized to provide accurate and timely information for blood gas analysis.
**Standard 13.3**: When priming with exogenous blood products, the use of prebypass ultrafiltration (PBUF) and/or washed red blood cells shall be used during the priming procedure.
**Standard 20.2**: The perfusionist shall collect data concerning the conduct of perfusion via a clinical registry or database to advance quality and safety.

Three new pediatric and congenital guidelines were introduced:

**Guideline 9.1**: The use of pH stat blood gas management strategy should be considered for neonates and infants undergoing hypothermia during cardiopulmonary bypass.
**Guideline 10.2**: Due to the higher metabolic demands of the pediatric patient, the perfusionist should consider higher blood flow rates to achieve adequate perfusion and DO_2_i requirements.
**Guideline 15.5**: Packed red blood cells should be washed via autotransfusion device or blood bank prior to transfusion whenever possible.

One additional pediatric and congenital standard was established:

**Standard 15.5**: The perfusionist shall consider the contribution of hemoglobin to patient DO_2_i and transfuse if indicated.

Further updates include the addition of base excess/deficit values and systemic vascular resistance (SVR) to the blood flow rate determination, the addition of age or weight-derived blood pressure goals, and consideration for blood prime compositions specifically: dilutional hematocrit, osmolarity/osmolality, matching current patient physiology, and inclusion of blood prime gas documentation. These additions were added due to their specific relevance to pediatric and congenital perfusion practice. A total of 31 new references were added from peer-reviewed medical journals.

## Discussion

The 2025 update of the *Standards and Guidelines for Pediatric Practice* reflects trends in the literature and environmental challenges experienced since 2019, specifically the COVID-19 pandemic. This revision emphasizes emergency preparedness, operative team communication and collaboration, and the development of institutional protocols for clinical and crisis management. Effective communication between the perfusionist and the operative team is a critical component of intraoperative care. Standard practices outlined in this revision include communicating deviations from intended treatment care plans and the use of an evidence-based and collaborative approach to minimizing blood loss peri-operatively. Conducting post-procedural debriefs are encouraged as are efforts to promote quality and safety through perfusion-specific data collection with participation in a database and/or registry.

Surveillance of critical clinical parameters on CPB was highlighted in this revision. Several clinical parameters were updated to be monitored continuously on CPB, including arterial oxygen saturation which was elevated to a standard. Access to point-of-care blood gas analyzers for timely and accurate evaluation was included as a standard. Additionally, consideration for DO_2_i was added due to the higher metabolic needs of the pediatric patient and the contribution of hemoglobin (standard) and higher flow rates (guideline) to maintain adequate DO_2_i. Perfusion management techniques specifically the treatment of exogenous blood with pre-bypass ultrafiltration and/or the use of washed packed red blood cells (pRBCs) during the priming sequence were deemed a standard in this revision. Guidelines specify washing pRBCs with cell salvage technology or blood bank technology prior to administration at other times during CPB. Consideration for pH-stat blood gas management strategy during hypothermia for neonates and infants was also stated. In total, five existing guidelines were elevated to standards and one new standard was introduced based on compelling evidence.

The *Standards and Guidelines for Pediatric Practice (2019)* were envisioned to be a “living document” and continuously reviewed at regular intervals. Following the 2025 update, the subcommittee anticipates a third revision in the next three to four years. This was the intent when reviewing the 2019 version, however, it was delayed due to the anticipated release of the adult *Standards and Guidelines for Perfusion Practice (2023)*. In practice guidelines-related publications, it is recommended that guidelines be reassessed for validity every three years, as nearly half of all guidelines are outdated after 6 years [10, 11]. Despite the delays faced in composing the current version, the timeline was consistent with those of other organizations such as the American Heart Association, with updates taking as long as 5–8 years until publication [12].

The review cycle for evaluating the *Standards and Guidelines for Pediatric Perfusion* should be brief as new data and techniques continue to evolve rapidly. Perfusion-specific registries such as The Perfusion Measures and Outcomes (PERForm) registry and the PediPERForm Learning Network Registry are also driving changes to perfusion practice [13]. These registries can ascertain discrete perfusion practices on cardiopulmonary bypass and “reflect adherence to evidence-based guidelines and professionally based standards and guidelines” [14]. Additionally, registries are collaborating to link patient data from the operating room to the intensive care unit. An example of this collaboration is PediPERForm and Pediatric Cardiac Critical Care Consortium (PC^4^) [13]. As registry data expands and inter-registry collaboration continues, identifying practice trends will become easier and may catalyze research and quality publications reinforcing the need for frequent review of the standards and guidelines. Going forward, the subcommittee plans to convene semi-annually to evaluate emerging topics for consideration during the formal review of the document.

The *Standards and Guidelines for Pediatric Perfusion (2025)* added multiple new standards and guidelines following a five-year period. Despite the inclusion of these references, conclusive evidence is lacking throughout much of the document. Limitations to this work include a lack of prospective randomized controlled studies, the use of surveys to evaluate practice trends, and a generalized lack of consensus among perfusionists regarding clinical practices. Additionally, given the small subcommittee, there is potential that the biases of the members affected the evidence evaluated and chosen. Moving forward, the next revision may include an improved system by which evidence is selected, evaluated, and ranked.

The absence of a multidisciplinary collaboration with congenital and pediatric cardiac surgeons, anesthesiologists, intensivists, and perfusionists may have limited this process. A collaborative approach could have enhanced the literature review process, breadth of information presented, and possibly offered a more comprehensive evaluation of contemporary science and clinical practices in the development of this revision. Similar guideline documents for perfusionists, such as the European Association for Cardio-Thoracic Surgery (EACTS) guidelines for European adult cardiac surgery, are developed in a multidisciplinary fashion consisting of perfusionists, surgeons, anesthesiologists, and intensivists collaborating to generate guidelines to guide clinical practice in adult cardiopulmonary bypass [15–17]. However, the EACTS document differs substantially in scope and scale from than *Standards and Guidelines for Pediatric Practice* document and matching this effort would entail a significant shift in goals and procedure established in previous revisions. However, endorsement from surgical peers was obtained from the Congenital Heart Surgeons’ Society (CHSS). Members of these organizations were not included in the subcommittee and therefore did not assist in the production of the revision or the assessment of the evidence. Opportunities to seek endorsements internationally may be possible with future revisions.

Perfusion practices are continually evolving with the introduction of new technology, devices, and clinical practices [9, 18]. Additionally, the exponential growth in medical knowledge coupled with an overwhelming volume of research publications poses significant challenges for clinicians striving to stay informed of current best practices [19, 20]. As such, the use of clinical practice guidelines has become an essential tool for clinicians as they are based on an exhaustive, systematic review of available literature and are composed by field experts. Additionally, published standards and guidelines not only keep clinicians apprised of the latest evidence but may also promote the standardization of practice techniques [21]. Updating the standards and guidelines ensures alignment with advancements in technology and research, strengthens the profession’s credibility, and may promote quality and consistency of care provided to pediatric patients undergoing cardiopulmonary bypass.

## Data Availability

The data supporting this work are publicly available and cited in the References section of this manuscript.
